# Importance of microenvironment to arbovirus vector distribution in an urban area, São Paulo, Brazil

**DOI:** 10.1590/0037-8682-0504-2019

**Published:** 2020-04-03

**Authors:** Marylene de Brito Arduino, Luis Filipe Mucci, Luciana Mamede dos Santos, Marilena Fogaça de Souza Soares

**Affiliations:** 1 Secretaria de Estado da Saúde. Superintendência de Controle de Endemias. Laboratório de Biologia e Ecologia de Culicídeos, Taubaté, SP, Brasil.; 2 Fundação de Ciência, Aplicações e Tecnologia Espaciais, São José do Campos, SP, Brasil.; 3 Secretaria de Estado da Saúde, Instituto Adolfo Lutz, Centro de Controle de Doenças, Regional Taubaté, Taubaté, SP, Brasil.

**Keywords:** Aedes aegypti, Aedes albopictus, Arbovirus disease, Vector control, Landscape ecology

## Abstract

**INTRODUCTION:**

The study of the landscape ecology, biological microhabitat, and epidemiological implications for the distribution of the main vectors *Aedes aegypti* and *Aedes albopictus* contribute to the prevention and control actions regarding the diseases they transmit.

**METHODS:**

This study sought to assess data on positive properties of the vector control program activities from 1998 to 2010. An entomological survey was also carried out on a sample of buildings collecting larvae and pupae from containers between October and April (spring / summer) from 2002 to 2005. We assessed the physico-chemical data of the water in 20% of positive containers. The vegetation and urbanization were assessed with the aid of satellite images and microenvironments were classified as urbanized, woods, and shrubs. The data were analyzed using statistical and geoprocessing software.

**RESULTS:**

*Ae. aegypti* and *Ae. albopictus* colonized all types of microhabitats and microenvironments, predominantly in the urbanized area, in isolation and in coexistence. The microhabitat of *Ae. aegypti* showed a temperature gradient greater than that of *Ae. albopictus*, and there was an association with urbanized areas for the first species and wooded areas for the last species.

**CONCLUSIONS:**

Landscape ecology and intra-urban differences favor different microclimates, which contribute to the coexistence of species in the urban environment in an area close to the forest, raising the risk of other arbovirus infections in urban areas. The ecological niche should be considered for *Ae. albopictus*. Entomological and virologic monitoring are suggested as arbovirus surveillance actions in urban infested centers near preserved forests.

## INTRODUCTION

Dengue is the oldest and most widespread arbovirus disease worldwide. Nearly half of the world’s population is at risk of dengue infection. Transmission occurs in almost all tropical, subtropical, and sub-Saharan African countries and, more recently, in European countries^1^.

Prior to 2000, only isolated outbreaks of chikungunya virus infection occurred in Africa and Asia. With the adaptation and territorial expansion of vectors, epidemics have been more frequent, reaching the Americas and Europe. Since its introduction in 2013, more than one million cases have been recorded in the Americas^2^.

Transmission of the Zika virus is more recent, with the largest outbreak beginning in Brazil and expanding rapidly to Central America and the Caribbean. Almost 140,000 cases were confirmed in Brazil between 2015 and 2018. Zika is an extremely serious and complex problem, resulting in post-infection complications, such as microcephaly and Guillain-Barré syndrome, causing the World Health Organization to consider it a threat to global public health^3^. 

In the last decade, the world has witnessed yellow fever epidemics on the African continent and in several Latin American countries. According to data from the Pan American Health Organization, between 2016-2019, countries and territories (Bolivia, Brazil, Colombia, French Guiana, and Peru) in the Americas reported cases of wild yellow fever. There were 2,024 confirmed human cases and 795 deaths. The major concern is urban transmission, a situation that can have serious consequences for humanity because a large part of the world’s population is not vaccinated^4^ ^,^ ^5^.

The re-emergence and expansion of these arbovirus diseases is mainly due to increased infestation with the two main vectors (the mosquitoes *Aedes aegypti* and *Aedes albopictus*), which is promoted by the global movement of people and by high population concentrations in urban centers^1^. *Ae. aegypti* is considered the main vector for most arbovirus diseases in urban environments. The species originated in tree hollows from wild areas of the African continent and later adapted to the urban environment, using a wide variety of artificial containers for reproduction. Today, it is found in many countries on almost all continents^6^.

*Ae. albopictus* originated in the forests of Southeast Asia and, since the 1980s, has infested America and Europe, currently reaching countries on all continents, except for Antarctica. Under laboratory conditions, it has competence for 22 different viruses^7^. *Ae. albopictus* is an urban vector of dengue virus in some parts of China, Seychelles, Japan, and Hawaii. In addition to dengue virus, 11 different viruses were isolated from field specimens, including yellow fever virus. In Europe, this mosquito is considered the main vector of chikungunya virus^2^ ^,^ ^7^ ^-^ ^11^.

Currently, forested areas and parks are bordered by urban spaces, i.e., without a transition zone. This typically occurs because of unplanned urban sprawl^12^. Examples include the São Paulo Forest Gardens, the Oswaldo Cruz Institute’s Botanical Garden in Rio de Janeiro, and many municipalities on the Brazilian coast that border the Atlantic Forest. Studies have reported the circulation of many viruses in this forest, such as those of Eastern equine encephalitis, Venezuelan equine encephalitis, Western equine encephalitis, St. Louis encephalitis, among others, whose agents can be transmitted by *Ae. aegypti* and *Ae. albopictus* ^13^ ^-^ ^15^.

The population densities of *Ae. aegypti* and *Ae. albopictus* may vary as a function of temperature and rainfall, blood meal, and reproduction, as well as proximity to vegetation or urban centers^16^. Therefore, quantifying the environmental requirements of these vectors can also help in the association with landscape epidemiology. In this sense, high-resolution environmental analysis allows assessing ecological niche factors and predicting species distributions, which has made geoprocessing technologies an important tool in decision-making toward improving the efficacy and cost-effectiveness of the control of arbovirus diseases^17^.

The present study assessed the spatial distribution of *Ae. aegypti* and *Ae. albopictus* in their breeding sites and the physico-chemical characteristics of the breeding sites in relation to intra-urban landscape differences. Understanding the distribution and ecology of these species can contribute to improving actions regarding the surveillance of arbovirus diseases whose agents are transmitted by these mosquitoes.

## METHODS

**FIGURE 1:**
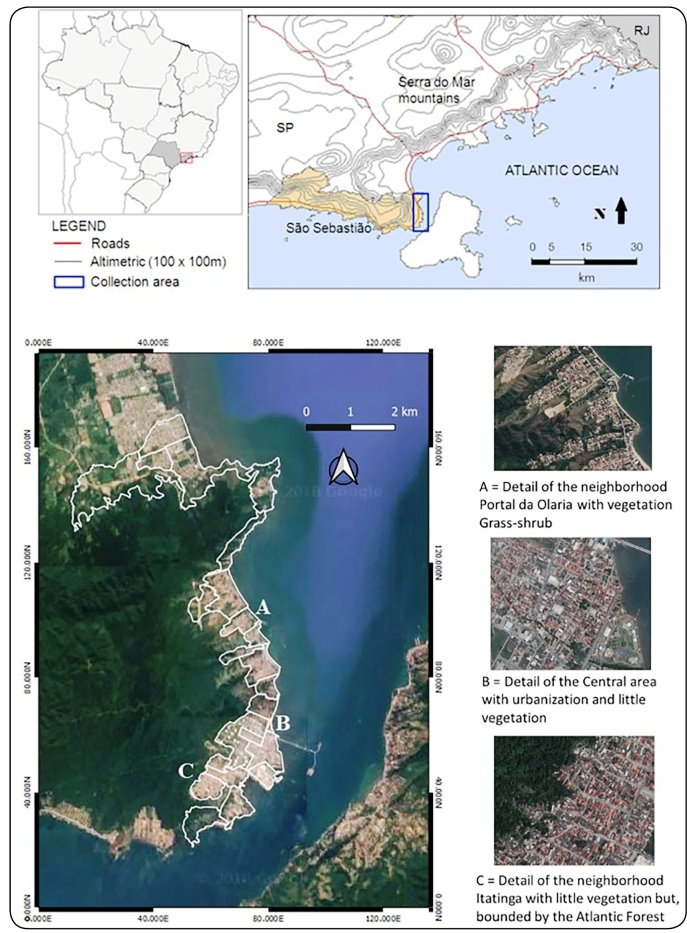
Study site detailing the intra-urban differences, São Sebastião, São Paulo, Brazil.

The present study was conducted in São Sebastião, a municipality on the northern coast of the state of São Paulo. The region is infested by both mosquito species and is a transmission site for the four dengue virus serotypes, in addition to Zika, chikungunya, and yellow fever^18^. The study site is the oldest and most urbanized part of the municipality, and the city center has old and historic buildings, with commercial properties, squares, and houses with wooded gardens. Next to the city center, there are densely populated neighborhoods with buildings very close to forested areas. The other neighborhoods have characteristic horizontal urbanization interspersed with gardens and abundant vegetation in backyards. In many sections, the urban grid penetrates areas of preserved vegetation, resulting in the lack of a transition zone (Figure 1).

The municipality is a tourist destination, with an urban population of 73,942 inhabitants, according to data from the Brazilian Institute of Geography and Statistics (Instituto Brasileiro de Geografia e Estatística - IBGE). The total territorial area of the municipality is 479 km^2^, with 27,000 hectares being part of Serra do Mar State Park, which is covered by dense ombrophilous forests^18^. The climate is rainy tropical with a mean rainfall below 60 mm in the driest month. The mean annual temperatures are not lower than 18ºC in the coldest months and above 30ºC in the warmer months^19^.

### Entomological data

Based on the results of the systematic activities of the Municipal Control of Dengue Vectors Program from 1998 to 2010, properties where *Ae. aegypti* and *Ae. albopictus* were found and where both species coexisted at the site were recorded. In addition to these data, entomological surveys were conducted from October 2002 to April 2003 and from October 2003 to April 2004, the months considered to have the highest vector reproduction rate in the region. The chosen sampling method has been used to survey entomological indicators. The property was the sampling unit, and the block was selected by systematic, random, and monthly sampling in a single stage, with substitution^20^.

All containers with water were inspected, and 100% of the pupae and samples of fourth instar larvae were collected. All specimens were identified to the species level at the Laboratory of Culicidae Biology and Ecology of the Superintendence of Endemic Control of the State Department of Health of São Paulo using a dichotomous key^21^.

In some of the positive containers (21%), the water volume, pH, conductivity, salinity, dissolved oxygen, and temperature of the water in the containers were measured in the field using a portable multi-parameter meter (Sension 156^TM^; Hach Company, USA).

Vegetation cover data were obtained from high-resolution images from the IKONOS^®^ satellite on image acquisition dates as close as possible to the breeding site surveys (between 2002 and 2004). The images were classified as follows: a) urbanized, corresponding to an urban environment with a predominance of buildings and paved streets; b) woods, representing an environment of arboreal vegetation and forest; this area consisted of wooded backyards, orchards, forest remnants, and preserved forests; and c) grass-shrub, considered a mixed-vegetation environment, including areas with grass cover and shrub vegetation in backyards, gardens, and public squares.

Each environmental class, i.e., urbanized, woods and grass-shrub, was defined by establishing a radius of 50 meters from the center of each positive property. After determining each class, the values were summed (100%) to define the percentage of each class for each breeding site occupation situation, i.e., species found alone or in coexistence. 

Each property with a positive container was considered a point. Properties where there were containers with *Ae. aegypti* alone and *Ae. albopictus* alone and/or with the two species coexisting were considered for each of these situations. However, to avoid data overlap, on properties where there was more than one positive container for one of the two species alone or in coexistence, only one positive container had been registered.

### Cartographic data

The distribution of properties and environmental data was represented on a street-level grid provided by the São Sebastião Municipal Government in digital format. Control points for geo-referencing and the geographic coordinates for each breeding site were obtained in the field using a Garmin^®^ Vista GPS receiver.

The properties were geocoded according to the registration number found in the municipality’s georeferenced database. The geographic database was built using Terra View 4.1 software. Maximum likelihood using the classification technique was applied to Quickbird (16/12/2002 and 03/03/2006) and IKONOS (11/04/2009) satellite images.

### Data analysis

The data for positive properties from the Control Program database were plotted on a simple map to show the presence of species per year, considering the situation in which the species were found at the breeding site alone or in coexistence throughout the 1998-2010 historical series.

Regarding the distribution data for the breeding sites collected in the 2002-2005 field surveys, the tabular data for the positive presence of immature life stages, microhabitat and macrohabitat variables, as well as vegetation cover, were stored in a database built using the program StatsDirect^®^, through which statistical tests of association were performed.

For the remote sensing data, Tasseled cap transformation was applied to the satellite images, resulting in a vegetation index image, only for the results from the 2002-2005 surveys, when physico-chemical data for the water from the breeding sites were collected. 

The Shapiro-Wilk and Levene tests were used to evaluate data normality and homoscedasticity, respectively. Most of the samples did not show a normal distribution, and there was no equality of variances. Therefore, nonparametric tests, including the Kruskal-Wallis test, were used to compare the proportions of *Ae. aegypti* and *Ae. albopictus* and both in coexistence as well as to compare the classes. Multiple comparisons were performed using the Dwass-Steel-Critchlow-Fligner (DSCF) test in the StatsDirect software.

## RESULTS

A total of 5,566 properties were surveyed during the recording of breeding sites. Of 15,879 containers with water, 1,642 contained culicids. Eleven genera and 30 species were identified from a total of 27,135 specimens. Of these, 44% (n = 11,955) were *Ae. aegypti*, 17.5% (n = 4,757) were *Ae. albopictus*, and 38.5% were of other species (n = 10,423).

The total number of positive containers was 839 and 591 for *Ae. aegypti* and *Ae. albopictus*, respectively. Each property with a positive container was considered a point, resulting in 376 breeding sites where *Ae. aegypti* was found alone, 192 breeding sites where *Ae. albopictus* was found alone, and 114 sites where the two species coexisted.

**FIGURE 2:**
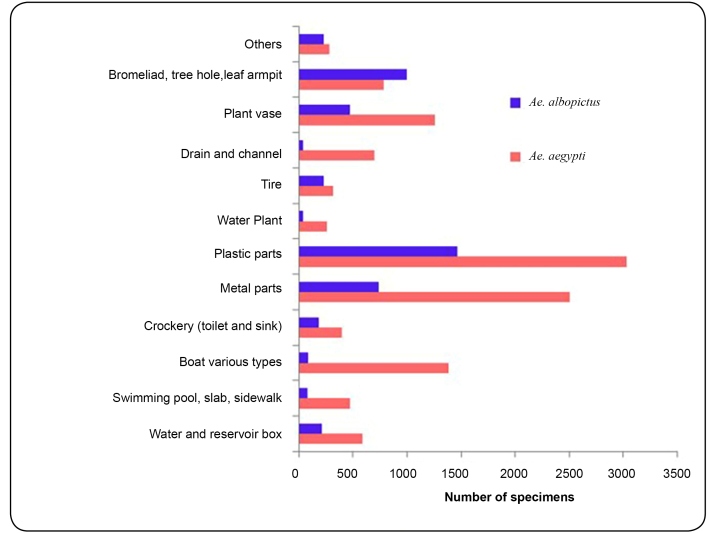
Distribution (number of specimens) of *Ae. aegypti* and *Ae. albopictus* per container type for the period between October 2002 and April 2004, São Sebastião, SP, Brazil.

The two species were found in all types of containers and the number of specimens of *Ae. aegypti* was less abundant in natural containers (Figure 2).

*Ae. aegypti* was found alone at 59% of the breeding sites; it coexisted with 17 species, and the most common coexistence was with two species, *Ae. albopictus* (21.6%) and *Limatus durhami* (6.9%), totaling 90.8% of the specimens collected. *Ae. albopictus* was found alone at only 26.2% of the breeding sites; it coexisted with 18 species, reaching 90.4% of the total collected in coexistence with five species, including 30.6% with *Ae. aegypti* and 21.3% with *Li. durhami*.

The temperature, pH, dissolved oxygen, conductivity, and salinity ranges were higher when *Ae. aegypti* was alone at the breeding sites than when it coexisted with other species. For *Ae. albopictus*, the ranges for these parameters were lower when alone than when in coexistence (Table 1).

**TABLE 1:** Variation in the physico-chemical parameters of the water from the breeding sites with *Ae. aegypti* alone, with *Ae. albopictus* alone, and with the two species in coexistence from October 2002 to April 2004, São Sebastião, São Paulo, Brazil.

Species/Situation	Range	Volume	Temp.	pH	Od	Conductivity	Salinity
		ml	(ºC)		(mg/L)	(µS/cm)	(%_o_)
Isolated	Maximum	10,000,000	35.9	10.3	9.4	22,400.00	13.5
*Ae. aegypti*	Minimum	7	18	4.5	0.2	0	0
n=189	C.V. (%)	734.9	9.7	11.9	52.1	262.1	313.5
Isolated	Maximum	700,000	33.1	8.3	9.4	5,120.00	2.8
*Ae. albopictus*	Minimum	10	21.4	4	0.5	4	0
n=52	C.V. (%)	422.8	9.4	14.4	66.2	195.4	251.9
*Ae. aegypti* +	Maximum	500,000	35.4	8.9	10.3	16,110.00	9.2
*Ae. albopictus*	Minimum	25	21.3	4.3	0.5	2.4	0
n=54	C.V. (%)	469.2	9.6	15.6	49.8	307	358.9

**Od:** dissolved oxygen; **mL:** microliters; **mg/L:** milligram per liter; **µS/cm:** microSiemens per centimeter; **‰:** parts per thousand; **CV:** coefficient of variation.

**FIGURE 3:**
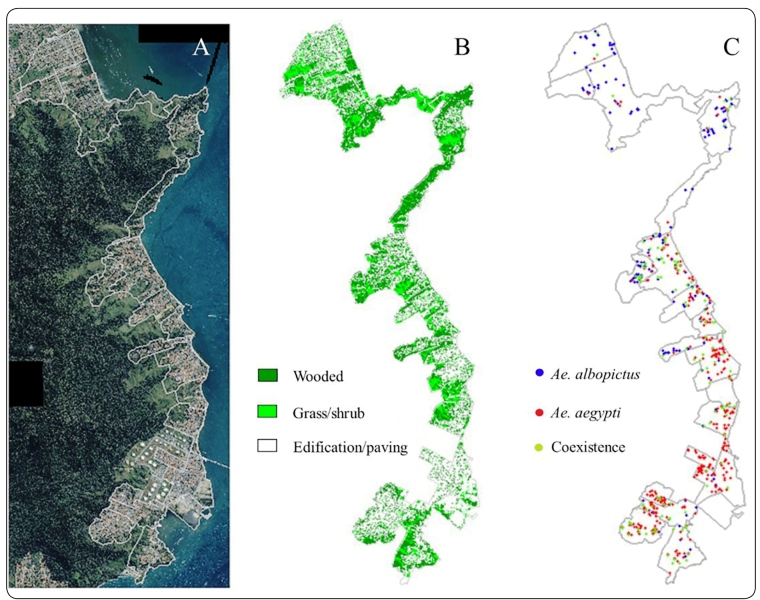
(A): Satellite image of the area showing the urban area and forest. (B): Proportions of urbanized, wooded and grass-shrub environments. (C): Distribution of locations with *Ae. aegypti* alone, with *Ae. albopictus* alone and with the species in coexistence from October 2002 to April 2004, São Sebastião, São Paulo, Brazil.

Figure 3 shows that highly urbanized areas are clearly surrounded by preserved forest and vegetation areas. The two species are spatially distributed in the entire study area, i.e., in all environments and in the three situations. However, *Ae. aegypti* was more concentrated in the urban environment, and *Ae. albopictus* was more concentrated in environments with more vegetation, even those in urban areas.

The proportional distribution of the positive locations in each environmental class for each species alone or in coexistence was similar: *Ae. aegypti* (70%, 20%, and 10%), *Ae. albopictus* (50%, 35%, and 15%), and coexistence (56%, 30%, and 14%) for the urbanized, woods, and grass-shrub classes, respectively. Both species were more abundant in the urban environment when they occurred alone and when they occupied the same breeding site. The environment with arboreal vegetation had the second largest number of breeding sites, followed by mixed-vegetation sites, for the three environments.

All tests showed significant differences between the containers with single species and in coexistence for the three environments (*H* test, p<0.0001). In the analysis of the proportion of breeding site locations for each species alone and in coexistence in relation to the environment, there was no significant difference between the breeding site locations in which *Ae. albopictus* was alone and breeding site locations where the species coexisted, except for the wooded environment (DSCF test, p<0.0001). 

**FIGURE 4:**
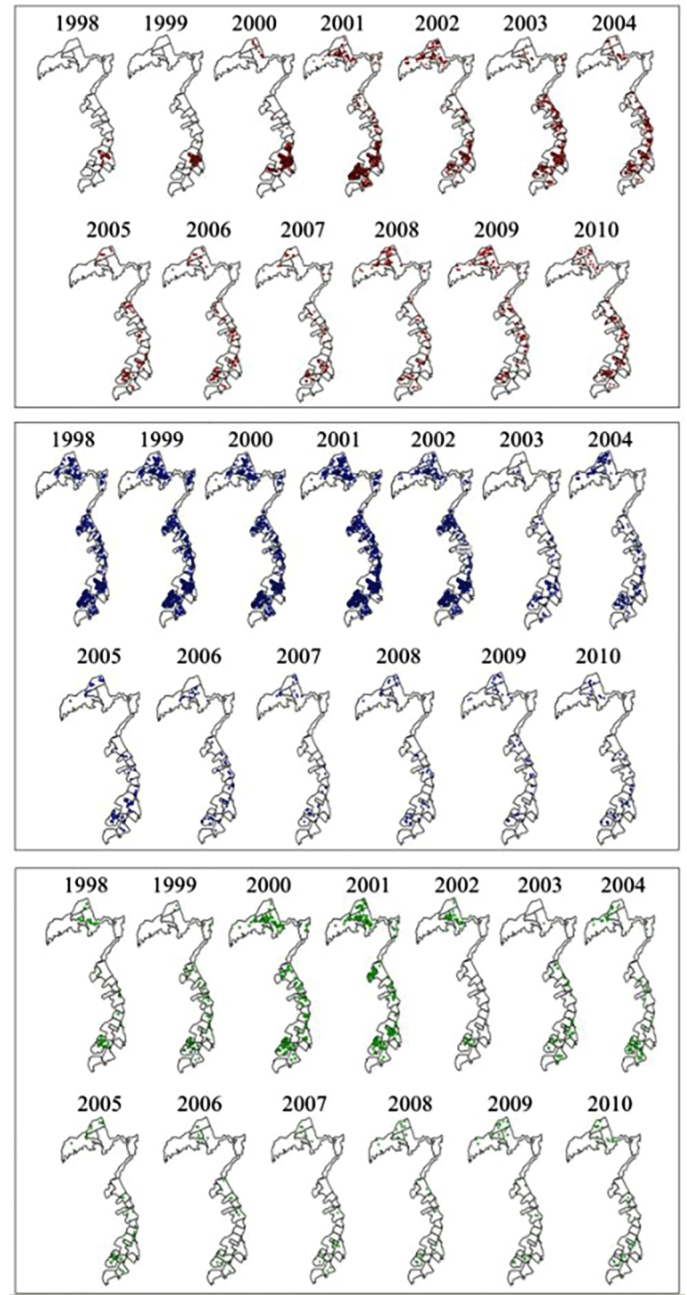
Distribution of positive properties for *Ae. aegypti* (red), *Ae. albopictus* (blue), and the two species in coexistence (green) from 1998 to 2010, São Sebastião, São Paulo, Brazil.

*Ae. albopictus* was initially the predominant species at the study site, but *Ae. aegypti* became the most frequent species over the years. However, both alone and in coexistence, *Ae. albopictus* was present throughout the entire study site in all years (Figure 4).

## DISCUSSION

The spread of cities to or near preserved areas occurs in many regions of Brazil. More than 200 arbovirus species have been isolated in the country, of which 40 viruses are pathogenic to humans and are maintained in the zoonotic cycle in various existing natural ecosystems^22^ ^-^ ^24^. Currently, Brazil is widely infested by the two mosquito species studied, posing a high risk for the urbanization of other viruses^1^ ^,^ ^25^.

In the region where the study was conducted, all municipalities are bordered by the Atlantic Forest or forest fragments, where arboviruses pathogenic to humans circulate^13^ ^,^ ^23^ ^,^ ^24^. The number of species found in the breeding sites and in coexistence shows that the area has characteristics of recent deforestation and is located close to the forest.

*Ae. aegypti* and *Ae. albopictus* developed in all types of breeding sites and environments. However, the former species was predominant, and the urban space had a higher frequency of both species, both when alone at a breeding site and when in coexistence. Much of the urban area ends immediately at the forest, i.e., the mosquito species occur very close to a preserved environment, increasing the likelihood that they will be involved in wild cycles and will then begin to transmit viruses in urban environments. Studies have reported and attempted to clarify the relationship of coexistence between *Ae. aegypti* and *Ae. albopictus*, with adaptive advantages found for one or the other species. However, there are many areas where they coexist, although one species predominates^16^ ^,^ ^26^.

It was possible to demonstrate that *Ae. aegypti* was strongly associated with urbanization, even in an urban environment surrounded by preserved forest. The occurrence of *Ae. albopictus* in environments with vegetation and more commonly coexisted with other species than did *Ae. aegypti.* These findings support the arguments that *Ae. aegypti* has a stronger association with urbanization and *Ae. albopictus* has a stronger association with vegetated environments^16^ ^,^ ^26^ ^,^ ^27^. Points with more containers where the two species coexisted were also concentrated in the urban environment. It is worth noting that the landscape of the studied municipality consists of properties with gardens and one-story houses even though it is highly urbanized.

The water in the containers in which *Ae. aegypti* was found alone showed greater variation in physico-chemical variables, and the temperature range was higher, suggesting that the location received greater insolation. These data agree with the results of studies that analyzed the land surface temperature (LST) and urban heat islands in the city of São Paulo and found that LST was the most influential factor in increasing the incidence of dengue^28^. In another study, the temperature in an environment with less vegetation and greater heat was associated with an increase in the breeding sites with *Ae. aegypti* ^29^.

Conversely, lower temperatures and a smaller range were recorded in water in the containers in which *Ae. albopictus* was found. Those that contained both species were in environments with intermediate temperatures. This suggests that the containers occupied by *Ae. albopictus* were located close to vegetation, even in highly urbanized environments.

Studies conducted in areas in which *Ae. aegypti* and *Ae. albopictus* coexisted indicate the influence of climatic factors. A greater abundance of the former was observed in environments with higher temperatures, and a greater frequency of the latter was observed in environments with greater rainfall. These studies use data from the region that do not reflect differences in microhabitat on a small scale. Although they appear subtle, these differences may determine, for example, the distribution, permanence, abundance, and coexistence of these species in each region^16^ ^,^ ^26^.

As already noted, the study site is close to a preserved forest. There is an abundance of vegetation in backyards and public squares, especially in neighborhoods farther from the urban center. Areas with a greater presence of vegetation have milder temperatures, while those with a predominance of buildings can have even higher temperatures, forming urban heat islands^28^ ^,^ ^29^. It is believed that due to these landscape features, different “microenvironments” form throughout the urban environment of São Sebastião.

These microenvironments have microclimates that are characterized by different water temperatures, air temperatures, relative humidity, evapotranspiration, and insolation gradients, among others. These factors compose the ecological niche of the microhabitat of each species, i.e., the microhabitat requirements that each species needs to develop and remain in the macrohabitat^30^.

 The landscape ecology and intra-urban differences allow the existence of several microclimates that can favor the permanence of both species, which explains the occupation of different types of breeding sites and the entire urban environment by both species. This is reinforced by the temperature gradient data for each species. Studies report that biotic and abiotic factors may favor the permanence and coexistence of species both in the adult and larval stages^16^ ^,^ ^26^ ^-^ ^31^.

The 1998-2010 historical series makes it clear that *Ae. albopictus* was predominant in the study site. With the arrival of *Ae. aegypti*, it was possible to observe that the latter, in addition to occupying the same breeding sites as the former, evidenced by the number of properties with containers containing both species, became the most frequent species in the study site. However, it is also possible to observe that *Ae. albopictus* remains throughout the entire urban area, although at a lower density, in all years.

These results reinforce the importance of the landscape and the influence of vegetation cover on the formation of different microclimates. One study conducted in southern Florida suggested that microclimatic variables contribute to the exclusion and coexistence of *Ae. aegypti* and *Ae. albopictus* in the area^26^.

The permanence of the two species in an urban environment close to forests increases the risk of occurrence and maintenance of arbovirus infections. A survey conducted in parks in the metropolitan region of São Paulo found that *Ae. aegypti* was present at the edge of the parks, whereas *Ae. albopictus* was found inside the parks^32^. In an investigation of a dengue outbreak in Tokyo, Japan, a population of *Ae. albopictus* that colonized a park in the city center was decisive for the transmission of dengue^8^.

In the northern part of the city of São Paulo, a study reported that *Ae. albopictus* coexisted with other wild species and arbovirus vectors in a preserved environment. *Ae. aegypti* was found in properties bordering forested areas^15^, a situation very similar to that found in our study. Shortly after the above report, the area was the target of yellow fever virus transmission. Although the cases occurred in a wild environment and with the involvement of another species, the presence of these species and the proximity of the forest increased the risk of urbanization of yellow fever in the city. The rapid expansion of transmission led the World Health Organization to declare areas that, for decades, had been free of the disease as areas of ​​endemic transmission, including the city of São Paulo^4^ ^,^ ^5^.

Recently, a global alternative strategy for the control of dengue was proposed, considering smaller geographic units and data on the spatial characteristics of areas infested with *Ae. aegypti* ^33^. Generally, control programs only focus on this species, which maintains habits almost exclusively associated with the anthropogenic environment. These programs do not consider biological aspects and the ecological niche of *Ae. albopictus*, discussed in this study, especially in areas infested by this species and close to natural environments.

The species maintains wild characteristics, occupies containers in vegetated areas and close to forests, frequently inhabits natural breeding sites and coexists with wild species. This species should be considered not only in the maintenance of the transmission of viruses that already circulate in the urban environment but also in the possible urbanization of other arboviruses existing in the Atlantic Forest and other forests in Brazil^14^ ^,^ ^15^ ^,^ ^22^ ^-^ ^24^.

Although the study was conducted during only part of the period, the results of our analysis clearly indicate that these species use different strategies that allow their permanence and coexistence in urban environments. It is necessary to consider the landscape aspects of the environment and the behavior of each species for their targeted and timely control. Such control measures include entomological and virologic monitoring in areas that are infested and close to preserved forests.

## References

[1] Ryan SJ, Carlson CJ, Mordecai EA, Johnson LR (2019). Global expansion and redistribution of Aedes-borne virus
transmission risk with climate change. PLoS Negl Trop Dis.

[2] Burt FJ, Chen W, Miner JJ, Lenschow DJ, Merits A, Schnettler E (2017). Chikungunya virus:an update on the biology and pathogenesis of
this emerging pathogen. Lancet Infect Dis.

[3] Alaniz AJ, Bacigalupo A, Cattan PE (2017). Spatial quantification of the world population potentially
exposed to Zika virus. Int J Epidemiol.

[4] World Health Organization (WHO) (2019). Yellow Fever.

[5] Ministério da Saúde (MS). Secretaria de Vigilância da Saúde (2019). Monitoramento de Febre Amarela no Brasil.

[6] Kraemer MUG Sinka ME, Duda KA Barker CM (2015). The global distribution of the arbovirus vectors Ae. aegypti and
Ae. albopictus. eLife.

[7] Gratz NG (2004). Critical review of the vector status of Ae.
albopictus. Med Vet Entomol.

[8] Kobayashi D, Murota K, Fujita R, Itokawa K, Kotaki A, Moi ML (2018). Dengue Virus Infection in Ae. albopictus during the 2014
autochthonous dengue outbreak in Tokyo Metropolis, Japan. Am J Trop Med Hyg.

[9] IEC detecta vírus da Febre Amarela em mosquito *Ae.
albopictus* no Brasil.

[10] Caminade C, Medlock JM., Ducheyne E, McIntyre KM, Leach S, Baylis M (2012). Suitability of European climate for the Asian tiger mosquito Ae.
albopictus: recent trends and future scenarios. J R Soc Interface.

[11] Li Y, Kamara F, Zhou G, Puthiyakunnon S, Li C, Liu Y (2014). Urbanization Increases Ae. albopictus Larval Habitats and
Accelerates Mosquito Development and Survivorship. PLoS Negl Trop Dis.

[12] Pedro LC (2011). Geomorfologia urbana: impactos no ambiente urbano decorrente da
forma de apropriação, ocupação do relevo. Revista Geografia em Questão.

[13] Lourenço-de-Oliveira R, Castro MG, Braks MAH, Lounibos LP (2004). The invasion of urban forest by dengue vectors in Rio de
Janeiro. J Vector Ecol.

[14] Romano-Lieber N, Iversson LB (2000). Inquérito soroepidemiológico para pesquisa de infecções por
arbovírus em moradores de reserva ecológica. Rev Saúde Pública.

[15] Mucci LF, Medeiros-Sousa AR, Ceretti-Júnior W, Fernandes A, Camargo AA, Evangelista E (2016). "Haemagogus leucocelaenus and Other Mosquitoes Potentially
Associated With Sylvatic Yellow Fever In Cantareira State Park In the São
Paulo Metropolitan Area, Brazil,". J Am Mosq Control Assoc.

[16] Bracks MAH, Honório NA, Lounibos LP, Lourenço-de-Oliveira R, Juliano AS (2004). Interspecific competition between two invasive species of
container mosquitoes, Ae. aegypti and Ae. albopictus (Diptera: Culicidae),
in Brazil. Ann Entomol Soc Am.

[17] Malone JB, Bergquist R, Martins M, Luvall JC (2019). Use of Geospatial Surveillance and Response Systems for
Vector-Borne Diseases in the Elimination Phase. Trop Med Infect Dis.

[18] Sistema de Informações Florestais do Estado de São Paulo
(SIFESP) (2010). Mapas Florestais do Estado de São Paulo Por Município.

[19] Setzer J (1966). Atlas Climático e Ecológico do Estado de São Paulo. Ed. São Paulo:
Comissão Interestadual da Bacia do Paraná-Uruguai em colaboração com as
centrais elétricas de SP (CESP).

[20] Alves MCGP, Silva NN (2001). Simplifying the sampling method for evaluating the larval density
of Aedes aegypti in São Paulo State, Brazil. Rev Saude Publica.

[21] Forattini OP (2002). Culicidologia médica: identificação, biologia e epidemiologia.

[22] Rosa APAT (2016). The history of Arbovirology at Instituto Evandro Chagas, Belém,
Pará, Brazil, from 1954 to 1998. Rev Pan-Amaz Saude.

[23] Possas C, Lourenço-de-Oliveira R, Tauil PL, Pinheiro FP, Pissinatti A, Cunha RV (2018). Yellow fever outbreak in Brazil: the puzzle of rapid viral spread
and challenges for immunization. Mem Inst Oswaldo Cruz.

[24] Iversson LB, Rosa APAT, Rosa JT (1980). Estudos sorológicos para pesquisa de anticorpos de arbovírus em
população humana da região do Vale do Ribeira: II - inquérito em pacientes
do Hospital Regional de Pariquera-Açú. Rev Saúde Pública.

[25] Secretaria de Estado da Saúde de São Paulo (2017). Diretrizes para prevenção e controle das arboviroses urbanas no estado
de São Paulo.

[26] Lounibos LP, O'Meara GF, Juliano SA, Nishimura N, Escher RL, Reiskind MH. (2010). Differential Survivorship of Invasive Mosquito Species in South
Florida Cemeteries: Do SiteSpecific Microclimates Explain Patterns of
Coexistence and Exclusion?. Ann Entomol Soc Am.

[27] Montagner FRG, Silva OS, Jahnke SM (2018). Mosquito species occurrence in association with landscape
composition in green urban areas. Braz J Biol.

[28] Araújo RV, Albertini MR, Costa-da-Silva AL, Suesdek L, Franceschi NCS, Bastos NM (2015). São Paulo urban heat islands have a higher incidence of dengue
than other urban areas. Braz J Infect Dis.

[29] Azevedo TS, Bourke BP, Piovezan R., Sallum MAM (2018). The influence of urban heat islands and socioeconomic factors on
the spatial distribution of Aedes aegypti larval habitats. Geospat Health.

[30] Forattini OP (2004). Ecologia Epidemiologia e Sociedade.

[31] Ayala D, Costantini C, Ose K, Kamdem GC, Antonio-Nkondjio C, Agbor J (2009). Habitat suitability and ecological niche profile of major malaria
vectors in Cameroon. Malar J.

[32] Carvalho GC, Ceretti-Junior W, Barrio Nuevo KM, Wilk-da-Silva R, Christe RO, Paula MB (2017). Composition and diversity of mosquitoes (Diptera: Culicidae) in
urban parks in the South region of the city of São Paulo,
Brazil. Biota Neotrop.

[33] Vanlerberghe V, Gómez-Dantés H, Vazquez-Prokopec G, Alexander N, Manrique-Saide P, Coelho G (2017). Changing paradigms in Aedes control: considering the spatial
heterogeneity of dengue transmission. Rev Panam Salud Publica.

